# Beyond No Blame: Practical Challenges of Conducting Maternal and Perinatal Death Reviews in Eastern Ethiopia

**DOI:** 10.9745/GHSP-D-19-00366

**Published:** 2020-06-30

**Authors:** Abera Kenay Tura, Sagni Girma Fage, Alexander Mohamed Ibrahim, Ahmed Mohamed, Redwan Ahmed, Tadesse Gure, Joost Zwart, Thomas van den Akker

**Affiliations:** a School of Nursing and Midwifery, College of Health and Medical Sciences, Haramaya University, Harar, Ethiopia.; b Department of Obstetrics and Gynaecology, University Medical Centre Groningen, University of Groningen, Groningen, Netherlands.; c Department of Obstetrics and Gynaecology, Hiwot Fana Specialized University Hospital, Harar, Ethiopia.; d Department of Paediatrics, Hiwot Fana Specialized University Hospital, Harar, Ethiopia.; e Department of Obstetrics and Gynaecology, Deventer Ziekenhuis, Deventer, Netherlands.; f Department of Obstetrics and Gynaecology, Leiden University Medical Centre, Leiden University, Leiden, Netherlands.; g Athena Institute, Vrije Universiteit Amsterdam, Amsterdam, Netherlands.

## Abstract

Lack of a professional body to address patients’ complaints regarding quality of health care and absence of clear medicolegal guidance hamper maternal death reviews in Ethiopia.

## Key Messages

- Performing effective maternal death reviews as part of the maternal death surveillance and response program has been hindered by challenges including poor attendance, defensive attitudes, and blame shifting.
- Reviews of maternal and perinatal deaths should be based on a “no blame” principle. Emphasis should be on learning lessons and health professionals should feel safe to discuss the circumstances surrounding death.
- Meaningful reduction in maternal mortality requires a depoliticizing paradigm shift, a professional body to address patients’ worries, and clear medicolegal guidance to encourage providers to identify care deficiencies.

## BACKGROUND

Although knowledge on the determinants and causes of maternal and perinatal deaths is well established and effective strategies to decrease such deaths exist and are in use, maternal and child mortality comprises the unfinished agenda of the Millennium Development Goals project.^1^ In 2017 alone, 295,000 maternal and 2.5 million neonatal deaths were reported globally, 99% of them in low- and middle- income countries and from preventable causes.^2^^,^^3^ A maternal and perinatal death review is one of the key recommended strategies to decrease maternal and perinatal mortality by identifying cases and collecting relevant information permitting an effective response to prevent future deaths.^4^ With the overall objective of guiding actions to eliminate preventable maternal mortality, by ensuring that every maternal death (both inside and outside facilities) is counted to assess progress and evaluate impact of interventions, maternal death surveillance and response (MDSR) is widely implemented in low- and middle-income countries.^5^

Despite its wide uptake guided by the World Health Organization (WHO), a review by Smith et al showed that MDSR is often inadequately institutionalized and the shift from the existing facility maternal death reviews to MDSR was not well addressed.^6^ Successfully institutionalizing the MDSR requires having strong political commitment, adequate financial support, a strong death identification and notification system, and an adequate legal framework, as well as realizing the “no shame, no blame” culture. Other factors that were reported to contribute to the MDSR’s success included having professional societies serve as drivers of the process, professionals’ readiness to serve as member of the committee or independent assessor, and strong support from national ministries or international agencies like WHO, United Nations Population Fund, or the United Nations Children’s Fund.^6^

Successfully institutionalizing the MDSR requires having strong political commitment, adequate financial support, adequate legal framework, and a “no shame, no blame” culture.

From the program’s beginning, there have been concerns that introducing the MDSR may have an adverse effect on existing maternal death reviews and threaten confidentiality because of its focus on creating change by improving accountability as opposed to only stressing local care improvements.^7^ Initiatives to instigate change through increasing accountability may become a source of blame in nonconducive or unsafe environments. In such environments, diverting accountability to factors out of control become the norm to avoid being blamed for poor outcomes.^8^ Such situations have been reported in several death review practices.^9^^–^^11^

Successful maternal death reviews require supportive political and policy environments, individual responsibility and ownership, a proactive institutional ethos, and promotion of learning as a crucial part of improving services and quality of care.^12^ In contrast, fear of blame, lack of knowledge and skills, inadequate resources, missing documentations, and lack of clear guidelines comprise barriers to conduct effective death reviews.^13^

Smith et al stated that successful implementation of MDSR requires building support for the process in the lower cadres of the health system.^14^ However, one of the challenges in the current MDSR implementation is its weak support at the lower-level facilities where the review process and identification of response are identified and implemented.^15^ Lower-level facilities were found to receive little support, and many review processes were directed toward avoiding conflicts through deflecting responsibility for adverse outcomes to factors out of control^8^—thereby affecting both the conclusion and the actions to be taken to avoid similar deaths in the future.

## MATERNAL DEATH REVIEWS IN ETHIOPIA

The practice of maternal death reviews in Ethiopia goes back to the 1980s when Kwast et al conducted a confidential enquiry of maternal deaths in Addis Ababa.^16^ Since then, only a few death review practices^17^ have been reported until the MDSR program was introduced in 2013.^18^ Ethiopia is one of the countries where the MDSR was initiated to accelerate the reduction in maternal mortality as promoted in the 2013 WHO global framework.^5^ Four annual reports of MDSR implementation have been produced to date.^19^

Over the years, similar themes have emerged (delays in decision to seek care, delays in reaching appropriate facility, or not receiving appropriate care in facilities), and direct obstetric conditions are still the predominant cause of maternal deaths.^16^^,^^17^^,^^20^ Shortages of supplies and equipment continue to be reported to contribute to a majority of the deaths. In a study from facilities in Northern Ethiopia, only slightly more than half of the facilities were practicing good quality death reviews and took proper action for identified problems.^15^ The Ethiopian MDSR, although reported as a success by some^21^ is characterized by low coverage (captured 7.4% of expected maternal deaths), low review (reviewed 8% of identified deaths), and low staff participation.^20^ Often, factors contributing to bad outcomes (deaths or delays) are stated to be the result of factors outside the control of local health workers and policy makers.^8^^,^^15^

In a recent qualitative study of MDSR implementation in Ethiopia, Melberg et al showed that highly politicizing maternal deaths, as indicated by the common slogan of “no mother should die while giving birth” everywhere hinders the identification, reporting, review, or assignment of causes to maternal deaths. Health workers and officers leading MDSRs are under so much pressure to meet the political requirement of zero maternal deaths that there is a strong incentive to substantially underreport.^8^

The MDSR system functions without a national confidential enquiry to generate and recommend detailed lessons for practice and merely collates reports that arise from the bottom up reporting to generate annual MDSR reports.^19^ Thus, a safe and just national system of confidential enquiries could be of added value. Countries, like Kenya and Malawi, that have similar MDSR implementation challenges, including inadequately using the maternal death review process, underreporting of maternal deaths, lack of information on the response measures taken after a maternal death audit, and poor data quality, implemented confidential enquiries as a backup.^10^^,^^11^ Confidential enquiries can improve the challenges seen in the MDSR by: (1) creating a pool of anonymous reviewers, who are not reviewing their own cases unlike MDSR, which will judge the practice against a set of standards; (2) having delinked cases from facilities, providers, or locations to minimize introducing biases or the fear of blame during the review process; and (3) generating national reports and recommendations that may influence practice of care or policy.

Given the increases in infrastructure and human capital in Ethiopia,^22^ improvements in the provision of care would be expected, thereby reducing the mortality and morbidity. Improving care should first focus on evaluating the quality of care after clients reach the facilities (delay 3) rather than on problems in the community and en route to the facilities.^23^ But, the existing practice seems to focus on the delays in deciding to seek care (delay 1) and delays in reaching care (delay 2), which are partly out of the control of reviewing health staff and facility and sometimes totally outside the realm of the health system.^15^ Previous reviews indicated that focusing on delay 1 and 2 could mask effective design of implementation by focusing more on outside factors.^24^^,^^25^ Such reviews would result in generating unrealistic recommendations as the factors are outside of their sphere of influence.

Improving care should first focus on evaluating the quality of care after clients reach the facilities.

## THE PRACTICAL CHALLENGES IN EASTERN ETHIOPIA

On paper, maternal death reviews have been conducted in Eastern Ethiopia as part of the national MDSR program.^18^^,^^20^ In practice, performing effective death reviews suffered from poor attendance and a defensive attitude among participants, looking for deficiencies in care before a woman reached the hospital. This resulted in commonplace observations such as “lack of antenatal care,” “lack of awareness about danger signs in the woman,” “late referral,” or “absent prereferral management,” which appear to transfer responsibility for bad outcomes to the woman and referring facilities.^20^^,^^26^^,^^27^ In addition, existing death reviews did not include perinatal deaths yet contrary to the change of MDSR to maternal and perinatal death surveillance and response. Such challenges of not adequately reviewing perinatal mortality has been reported previously.^15^^,^^28^ Given the high burden of maternal mortality in many low-resource countries, effective perinatal death review will not be within reach in a shorter time.^15^

Moreover, learning during the audit was hampered by political sensitivities surrounding maternal death—a desire for no maternal deaths by political leaders and higher officials in hospitals or health bureaus.^8^ No or few maternal deaths were reported to occur in referring hospitals and health centers, while “(near-) death on arrival” was observed to be a common problem in the majority of the referral hospitals. At the lower-facility level, a woman in very critical condition sometimes appeared to be referred out immediately, sometimes without starting prereferral life-supporting care, out of fear that she would die at that facility. Such women will be registered by receiving facilities as “death on arrival.” These deaths will mostly not be reviewed by the receiving hospitals’ death review committee since they are thought to be the result of deficient care at the community level or at the level of referring facilities or because available information and documentation were insufficient for conducting a death review. Moreover, at the lower facilities, these deaths subsequently end up being forgotten since the referring facility will not perform a death review—but are reported as referred cases—and will mostly not even learn that referred women had passed away due to absence of appropriate feedback from higher to lower levels of care.^29^

Reviews of maternal and perinatal deaths should be based on a “no blame” principle.^4^^,^^30^ Emphasis should be on learning lessons, and health professionals should feel safe to discuss the circumstances surrounding each death.^4^ To improve the quality of maternal death reviews, the averting maternal and neonatal morbidity and mortality through obstetric audit (AMAN-MAMA) project was started in 2018 in selected hospitals in Eastern Ethiopia. As part of this program, 2 maternal and perinatal death review sessions were conducted, one in September 2018 and a second one in January 2019. The sessions were well attended by staff from all cadres from hospital management to health center staff, as well as experienced international assessors, all participants showed great motivation from all participants to contribute to case assessments, and learning and self-reflection were emphasized. During both sessions, unforeseen incidents hampered an effective audit process and forced the authors to consider that a “no blame” attitude during a maternal death review now collides with daily realities in Ethiopian life.

In September 2018, on the third day of the organized sessions, an attendee—a staff member at a neonatal care unit—did not show up to the session because he had been blamed and jailed for a pediatric death. He was later released from custody and charges were dropped. In addition, during the second maternal death review meeting held in one of the hospitals, a police officer, who was the partner of a woman receiving care, attacked the attending physician in the labor ward because he did not want his wife to give her informed consent for a cesarean delivery to be performed. His attack turned the hospital grounds into a violent scene with groups of people attacking one another, thus the circumstances for the maternal review session suddenly changed from ideal to impossible. These incidents illustrate how the realities in many low- and middle- income countries, such as lack of infrastructure to assess professional conduct and sudden interference by the public or police, may hamper feelings of safety to discuss bad outcomes among health professionals.

In a country on its long way to become a democratic state, civil unrest, lack of a professional body to address patients’ worries regarding quality of health care, and absence of clear medicolegal guidance in general^31^ hamper professionals to come forward and identify care deficiencies. Such factors comprise a threat to the advances that are being made in Ethiopia and many other emerging economies at present in improving pregnancy outcomes for their populations.^32^ There should be an agreement on the best societal outcomes and collaboration between public health and law enforcements to improve health.^33^ These scenarios underline the importance of addressing medicolegal aspects of death reviews both in principle and practice.^31^

## THE WAY FORWARD

Although ending preventable maternal mortality requires political priority,^34^ direct interference in the health system may not accelerate progress and may even deter the situation.^8^ In a country where maternal mortality is a highly political phenomenon,^8^^,^^35^ meaningful reduction in maternal mortality requires a depoliticizing paradigm shift. Health care providers should work within a conducive environment that enables them to thoroughly evaluate the pathways to death and generate lessons using their own perspectives, rather than using the narrow political lens of achieving the “no mother should die while giving birth” slogan everywhere and every time.

## Notes

**Peer Reviewed**

**First published online:** May 27, 2020

**Funding:** The maternal and perinatal death review was funded by Laerdal Foundation (ref no: 40241) as part of the AMAN-MAMA project.**Competing interests:** None declared.
